# Characteristics, disease burden and costs of COPD patients in the two years following initiation of long-acting bronchodilators in UK primary care

**DOI:** 10.1186/s12931-015-0295-2

**Published:** 2015-11-16

**Authors:** Yogesh Suresh Punekar, Sarah H Landis, Keele Wurst, Hoa Le

**Affiliations:** Value Evidence and Outcomes, GlaxoSmithKline Stockley Park, Uxbridge, UB11 1BT UK; Worldwide Epidemiology, GlaxoSmithKline R&D, Uxbridge, UK; Former employee of GlaxoSmithKline R&D, RTP, Durham, NC USA; Former employee of Parexel International, RTP, Durham, NC USA

**Keywords:** COPD, Costs, Long-acting bronchodilators, Primary care, Resource use, Retrospective cohort study, UK

## Abstract

**Background:**

To assess the symptomatic and cost burden among patients initiating long-acting bronchodilator (LABD) therapy and impact of adherence on healthcare resource use and costs.

**Methods:**

This retrospective cohort study identified patients with COPD who were newly prescribed a LABD (long-acting muscarinic antagonist [LAMA], long-acting beta_2_-agonist [LABA], a combination of LABA+LAMA or combination of LABA with inhaled corticosteroid [ICS]/LABA) between January 1, 2009 and November 30, 2013 from the UK Clinical Practice Research Datalink. Health care resource use, costs and symptom burden up to 24 months after treatment initiation were estimated. Adherence in the follow-up period was assessed using the medication possession ratio (MPR ≥80 %).

**Results:**

The cohort comprised 8283 LABD initiators (16 % LABA, 81 % LAMA and 3 % LABA+LAMA) and 9246 LABA+ICS initiators with generally similar baseline characteristics; prior exacerbation rate was higher in the LABA+ICS cohort. Less than half the patients (LAMA:42 %; LABA:34 % and LABA+ICS:34 %) were adherent to their index medication. Among adherent patients, the total annual per patient cost of COPD was £3008 for LAMA initiators, £2783 for LABA initiators and £3376 for LABA+ICS initiators; primarily due to general practitioner interactions. Among patients with a Medical Research Council dyspnea score recorded during 24 months follow-up, a substantial proportion of adherent patients (LAMA: 41 %; LABA: 45 %; LABA+ICS 44 %) had clinically significant dyspnoea (MRC ≥ 3).

**Conclusion:**

Cost and symptomatic burden of COPD was high among patients initiating maintenance treatment, including patients adherent with their initial treatment. General practitioner interactions were the primary driver of costs. Further, real world studies are required to address unmet needs and optimize treatment pathways to improve COPD symptom burden and outcomes.

## Background

Chronic obstructive pulmonary disease (COPD) is a preventable and treatable disease characterised by progressive and persistent airflow obstruction. COPD exacerbations and the comorbid nature of the disease pose a significant and increasing economic and social burden [1].

Inhaled long-acting bronchodilators (LABDs) with or without inhaled corticosteroids (ICS) are the mainstay of COPD therapy when symptoms persist, despite the use of short-acting bronchodilators (SABDs) [1, 2]. Two main classes of inhaled LABDs are long-acting beta_2_-agonists (LABAs) and long-acting muscarinic antagonists (LAMAs) which are occasionally supplemented by the oral LABD, theophyllines. The UK National Institute for Health and Care Excellence (NICE) guidelines recommend LABA or LAMA for patients with forced expiratory volume in 1 second (FEV_1_) ≥ 50 % of the predicted value, LABA+ICS or LAMA for those with FEV_1_ < 50 % of the predicted value, and in those with higher risk for exacerbations and persistent symptoms open triple therapy (LABA+ICS+LAMA) [2]. These recommendations are broadly in line with the Global Initiative for Chronic Obstructive Lung Disease (GOLD) strategy [1]. Comparative effectiveness studies suggest that there is potential for modest differences in efficacy between the inhaled LABD classes, but nonetheless each has shown potential to improve lung function, improve quality of life and reduce exacerbations [3].

Increasing evidence suggests that despite the availability of guidelines and LABD therapy, COPD patients suffer a high level of morbidity [4–8]. Results from an observational longitudinal cohort study in primary care indicated that a significant proportion of patients were symptomatic on treatment with a single bronchodilator [9]. Although poor symptom control and exacerbations lead to increased costs [10–13], appropriate use of maintenance therapy has the potential to reduce the overall costs of COPD management [13].

Furthermore, there is evidence to suggest that treatment outcomes are better and healthcare costs are lower among patients adherent with their prescribed medications [14]. Studies in patients with COPD have reported that adherence to maintenance therapy is generally low [15, 16]. Identifying and characterising patients who are adherent with their prescribed treatment, as well as the impact of adherence on health care resource use and costs, could inform the design of programs and interventions to improve the effectiveness of healthcare delivery. Therefore, this study aimed to assess the symptomatic and cost burden among patients initiating LABD therapy and estimate the impact of adherence on healthcare resource use and costs in the 24 months after LABD alone or LABA+ICS initiation.

## Material and methods

### Study design

This retrospective cohort study identified and evaluated patients with COPD who were newly prescribed LABD alone (LAMA, LABA, LABA+LAMA) or LABA+ICS in the UK Clinical Practice Research Datalink (CPRD). The database contains longitudinal data on patient characteristics; medical history (including records of referrals to consultants and hospitalisations); and treatment history [17]. The electronic records were anonymised, and the protocol was approved by the CPRD Independent Scientific Advisory Committee (ISAC protocol 13_073A2).

**Fig. 1:**
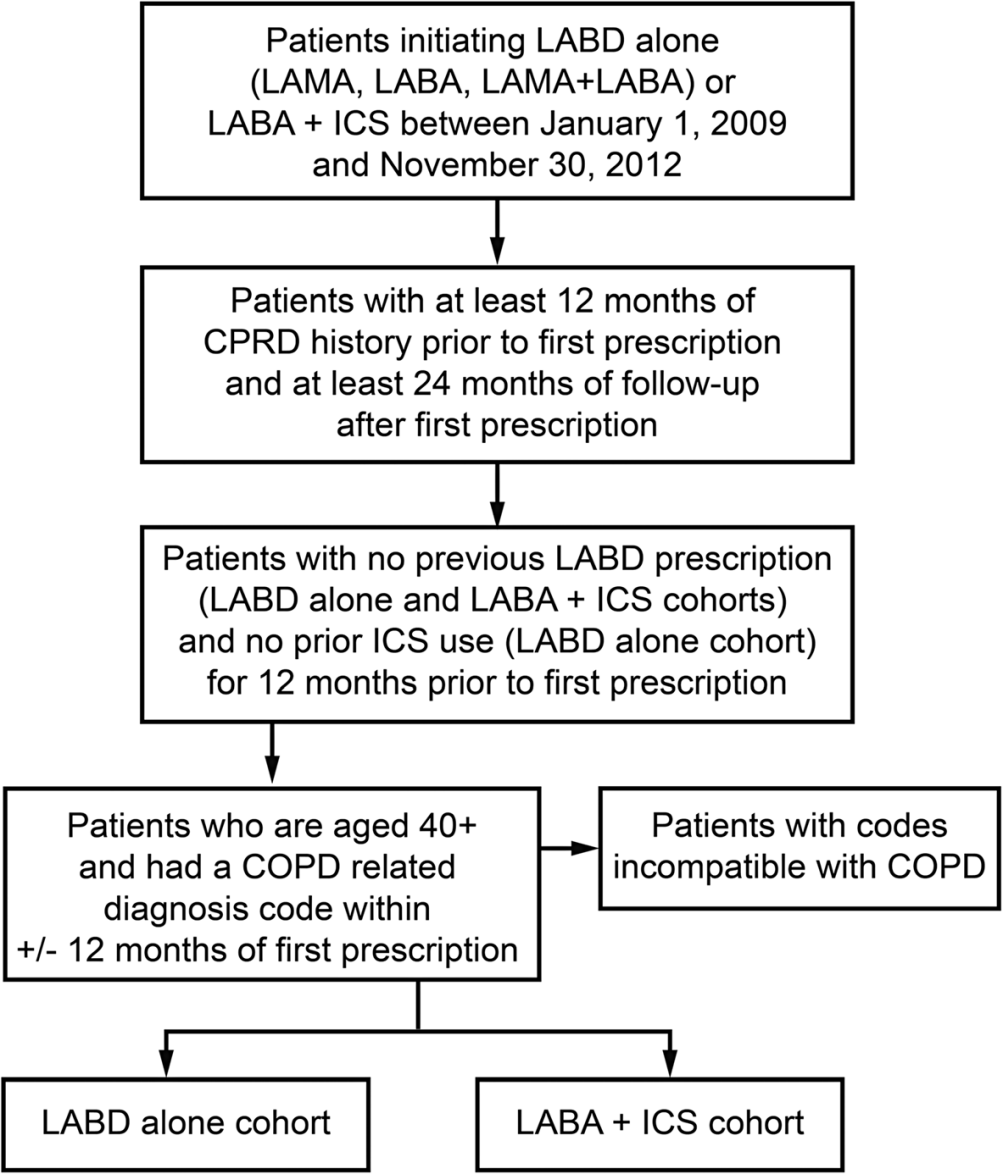
Cohort development algorithm. Algorithm to identify patients for inclusion in the current retrospective cohort study from the CPRD database. COPD, chronic obstructive pulmonary disease; LABA, long-acting beta2-agonist; LAMA, long acting muscarinic antagonist; LABD, long-acting bronchodilator; ICS, inhaled corticosteroid

Patients newly initiating a LAMA (single device), LABA (single device), LABA+LAMA (two devices) or LABA+ICS (single or two devices) between January 1, 2009 and November 30, 2013 were identified, and the date of the first relevant prescription was set as the index date (Fig. 1). For patients whose first prescription was LAMA or LABA, the patient record was searched for other COPD medications (LABD from a different class or ICS) which overlapped with the index LAMA or LABA by at least one day, and these were labelled as dual therapy of LABA+LAMA or LABA+ICS (concurrent treatment of ICS/LAMA was excluded because this combination is not licensed in COPD). Each medication category was mutually exclusive. Patients in the LAMA, LABA, and LABA+LAMA cohorts could not have received an ICS within 12 months prior to being included in the cohort. Use of a SABD was allowed in all groups. A prescription length of 30 days was used for all medications, irrespective of them having a recoded value for script length (<1 % had a value recorded).

Patients with at least one COPD ‘definite’ diagnostic code within 12 months (+/−) of the index date were included. Additional inclusion criteria were age ≥ 40 years and at least 24 months of follow-up from the index date (unless death occurred within 2 years of index date). Patients with an occurrence of a medical code for a condition that was incompatible with COPD diagnosis, such as lung or bronchial developmental anomalies, degenerative processes (cystic fibrosis or pulmonary fibrosis), bronchiectasis, pulmonary resection or significant respiratory disorders other than COPD (but excluding cancer) which could interfere with clinical COPD diagnosis or substantially alter the natural history of the disease, were excluded.

### Baseline patient and disease characteristics

Information was extracted on age, gender, body mass index (BMI), smoking status, Medical Research Council (MRC) dyspnoea scale scores and spirometry results from patient medical records. These patient and disease characteristics were described during baseline (value closest to index date which occurred at any time in the previous 12 months). Prior diagnosed comorbid conditions (according to the Charlson Co-morbidity Index) [18] were defined if the disease occurred at any time in the patient history up to the index date. COPD exacerbations during the 12 months prior to index date were defined as ‘severe’ episodes if resulting in a hospitalisation or emergency room visit for COPD or ‘moderate’ episodes characterised by management with COPD-specific antibiotics combined with oral corticosteroids (OCS) and/or medical diagnosis of COPD exacerbations outside hospital. All non-COPD hospitalisations were also captured. Exacerbations and non-COPD hospitalisations were expressed as annual rates per person-years with accompanying 95 % confidence interval [CI]). All cause general practitioner (GP) interactions during the 12 months prior to index date were captured and further classified into in-person GP (surgery) visits, administrative contacts, surgery correspondence, visits to a surgery nurse, out-of-office GP visits or GP home visits.

### Adherence to therapy

Treatment adherence to index medication in the 24 months of follow-up was evaluated using the Medication Possession Ratio (MPR). The LABA+LAMA cohort was excluded from the adherence analysis due to small sample size. Patients had to have at least two prescriptions for the index medication to be included in this analysis. Adherence was evaluated during total treatment time on the index medication, defined as the duration from the index date up to the date of the final prescription in the 24 months or before the first treatment change (switch to or addition of another maintenance treatment). MPR was calculated by adding the number of days supplied for all but the last prescription divided by the total treatment time (each patient had a unique denominator) [19]. MPR was expressed as a percentage, with non-adherence defined as MPR < 80 % and adherence defined as MPR ≥ 80 % [20]. Patient and disease characteristics as described above were also described during follow-up by adherence group, selecting the value closest to last prescription used in the MPR calculation (end of total treatment time). Airflow limitation during follow-up was not described as these data were missing for three quarters or more users regardless of adherence category. For this analysis, exacerbations and non-COPD hospitalisations were expressed as rates per person-years (95 % Confidence Internal (CI)) with a denominator of total person-time from the index date until censoring at death, last prescription used in the MPR calculation, or end of 24 months (if the patient never changed treatment).

### Predictors of resource use

To understand drivers of health care resource use, we fit predictive proportional hazard models for two dependent variables: 1) time to first moderate-severe exacerbation and 2) time to first severe exacerbation as proxy measures for health care resource use. Potential predictor variables included adherence (MPR <80 % vs >80 %), baseline demographic and clinical characteristics (age, gender, BMI, asthma co-morbidity, airflow limitation [Stage I-IV]) and resource use prior to LABD initiation (number of moderate to severe exacerbation [0, 1, ≥2], number of GP visits, number of non-COPD related hospitalisations [0, 1, ≥2], ICS use [Yes/No] and SABD use [≥4 vs <4]). Only variables that were significant at the alpha = 0.05 level were retained in the final model.

### Costs

The costs were expressed as mean annualised costs and were calculated using 2014 data (Table 1). The cost of a severe exacerbation was estimated using the National Health Service (NHS) reference costs 2013-14 (Healthcare Resource Group (HRG) codes: DZ21A-K for short and long stays) further weighted by the reported annual number of episodes from the cost schedules [21]. In addition, it was assumed that 66 % of patients would be admitted through Accident and Emergency (A&E) and 57 % of the patients would arrive at the hospital by ambulance, based on a clinical audit of COPD exacerbations in 2008 [22]. Therefore, 66 % of the HRG cost for A&E admission (HRG codes: T01A-04NA) and 57 % of HRG costs of ambulance transportation (code ASS02) was added to the weighted COPD HRG costs. The cost of a moderate exacerbation was compiled based on resource use as stated in the GOLD Strategy document [1] which was calculated using the NHS reference costs [21], the Personal Social Services Research Unit (PSSRU) 2014 [23], and the British National Formulary 65 (BNF 65) [22]. This included a GP consultation lasting for 11.7 minutes, an A&E visit with no admission (in 29 % of the cases), and a prescription of prednisolone (30 mg) and co-amoxiclav (500 mg). The cost of a non-COPD hospitalisation was estimated using a weighted average of short- and long-stay hospitalisation episodes from the PSSRU costs [23].

**Table 1:** Unit costs estimated for the resource use categories

Resource use item	Unit cost	Reference
Moderate exacerbation	£87.39	NHS reference costs 2013-14; PSSRU 2014
Severe exacerbation	£1,486.99	NHS reference costs 2013-14
Hospital episode	£1,806.50	NHS reference costs 2013-14
GP visit at surgery	£46	PSSRU 2014
GP visit out of office	£91	Assumed to be same as home visits
GP visit admin	£28	PSSRU 2014
GP visit correspondence	£3	PSSRU 2014
GP visit practice nurse	£13	PSSRU 2014
GP visit home visit	£91	PSSRU 2014
SABD	£3.30	PCA 2014
Theophylline	£3.90	PCA 2014
LAMA	£38.72	PCA 2014
LABA	£35.81	PCA 2014
LABA+ICS	£48.64	PCA 2014

GP, general practitioner; ICS, inhaled corticosteroid; LABA, long acting beta agonist; LAMA, long acting muscarinic antagonist; NHS, National Health Service; PCA, Prescription cost analysis; PSSRU, Personal Social Services Research Unit; SABD, Short acting bronchodilator

The costs of a GP visit in surgery and a GP visit at home were estimated to be £46 and £91, respectively, based on PSSRU costs [23]. The cost of an out-of-office GP visit was assumed to be equivalent to a GP home visit, and the cost of a GP administrative contact (£28) was assumed to be equivalent to a phone consultation at a GP surgery. The cost of a nurse visit at a GP practice was estimated to be £13 based on a typical 15.5 minute face to face consultation, and the cost of a GP correspondence was estimated to be £3 based on 30 % indirect costs of a typical non-face to face consultation [23]. Costs of medications were obtained from Prescription Cost Analysis 2014 (PCA) which provides details of number of dispensed items and net ingredient costs of all the prescriptions in England [24]. These were calculated at the BNF therapeutic class level (Table 1).

Resource use for each LABD cohort was estimated as mean number of events over the follow-up period. As each patient had a different follow-up time (until 24 months, treatment change or death), an annual event rate was calculated by dividing by the mean duration of treatment. Mean total annual costs were then estimated by multiplying the annual event rates with unit costs. These were estimated for all patients and separately for adherent and non-adherent patients.

## Results

### Patient disposition and demographic characteristics

The LABD alone cohort (81% LAMA, 16% LABA, and 3% LABA+LAMA) comprised 8283 patients with similar age distributions (mean [standard deviation (SD)], 69.2 [10.4] years), gender ratio (men:women, 56:44) and BMI (27.3 [6.1] kg/m^2^) across all prescription categories. Of these, 86 % had been diagnosed with COPD prior to initiating their LABD treatment whilst the remaining were diagnosed within 12 months after index prescription. The LABA+ICS cohort comprised 9246 patients with a mean [SD] age of 68.4 [11.4] years, gender ratio (men:women), 52:48, and BMI 27.7 [6.5] kg/m^2^); 75 % of patients having a prior diagnosis of COPD in their records. The proportion of COPD patients with one or more concomitant asthma codes was 13-16 % in the LABD alone cohort and 38 % in the LABA+ICS cohort. Demographic characteristics and patient distribution across all prescription categories are presented in Table 2.

**Table 2:** Patient demographics in 12 months prior to initiation of LABD or LABA+ICS

	LABD			
	LABA	LAMA	LABA+LAMA	LABA+ICS
	(*N* = 1317)	(*N* = 6695)	(*N* = 271)	(*N* = 9246)
Gender, male (%)	53	57	60	52
Age at index date, Mean (SD)	68.6 (10.8)	69.3 (10.4)	68.2 (9.8)	68.4 (11.4)
Comorbidity (%)^a^				
Cancer	9	10	10	10
Congestive heart disease	5	6	4	5
Dementia	1	1	0	1
Diabetes	13	13	11	13
Peripheral vascular disease	9	9	8	7
Renal disease	17	17	14	16
Anxiety	17	17	11	17
Asthma	16	13	15	38
Depression	16	14	14	14
Heart failure	6	7	4	7
Myocardial infarction	8	10	8	8
Stroke	5	6	3	5
Mean CCI (SD)	1.5 (0.9)	1.6 (0.9)	1.3 (0.7)	1.5 (0.9)
Smoking status (%)^b^				
Ex-smoker + never smoker	50	48	52	52
Current smoker	42	47	40	38
Unknown	8	6	8	10
BMI, Mean (SD)^bc^	27.8 (6.0)	27.2 (6.2)	27.5 (5.7)	27.7 (6.5)
Dyspnea (%)^b^ ^d^				
MRC < 3	59	55	48	57
MRC ≥ 3	41	45	52	43
Airflow limitation (%)^be^				
FEV1 ≥ 80 %	15	12	10	14
FEV1 50-79 %	64	61	64	52
FEV1 30-49 %	19	25	22	27
FEV1 < 30 %	2	3	5	4
Disease burden [rate per patient year (95 % CI)]^f^				
Any exacerbations (Moderate to severe)	0.49 (0.45-0.53)	0.49 (0.47-0.51)	0.47 (0.39-0.56)	0.70 (0.68-0.71)
Hospitalised (severe) exacerbations	0.07 (0.06-0.08)	0.09 (0.08-0.10)	0.10 (0.07-0.14)	0.12 (0.12-0.13)
Non COPD hospitalizations	0.36 (0.33-0.40)	0.39 (0.37-0.40)	0.39 (0.32-0.47)	0.42 (0.40-0.43)
Patients with more than 4 SABD prescriptions (%)	25	20	21	29

CCI, Charlson Comorbidity Index; COPD, chronic obstructive pulmonary disease; ICS, inhaled corticosteroids; LABA, long-acting beta2-agonist; LABD, long-acting bronchodilator; LAMA, long-acting muscarinic antogonist; MRC, Medical Research Council dyspnea scale 1-5; FEV1, Forced expiratory volume; SD, standard deviation; SABD, short-acting bronchodilator

^a^Charlson Comorbidity Index score calculated based on co-morbidities recorded any time in the patient history up until the index date

^b^Value closest to index date in prior 12 months

^c^Percentages represent distribution in patients with a known value. BMI data unknown for *N* = 1866 (28 %) LAMA users, *N* = 378 (29 %) LABA users, *N* = 77 (28 %) LABA+LAMA users and *N* = 3188 (34 %) of LABA+ICS users

^d^Percentages represent distribution in patients with a known value. Dyspnea data unknown for *N* = 2356 (35 %) LAMA users, *N* = 443 (34 %) LABA users, *N* = 99 (37 %) LABA+LAMA users and *N* = 4196 (45 %) of LABA+ICS users

^e^Percentages represent distribution in patients with a known value. Airflow limitation data unknown for *N* = 2359 (35 %) LAMA users, *N* = 450 (34 %) LABA users, *N* = 87 (32 %) LABA+LAMA users and *N* = 4340 (47 %) of LABA+ICS users

^f^Exacerbation rate during the full period of 12 months prior to index date

While patients initiating LABA+ICS had a higher rate of any exacerbations in the 12 months prior to the index prescription, less than half the patients in both group (LABD alone [35 %] and LABA+ICS [45 %]) had experienced at least one moderate or severe exacerbation (Table 2). The corresponding estimates for severe exacerbations were 8 % and 11 %, respectively. The frequency of non-COPD hospitalisations in the prior 12 months was similar between the two cohorts with 21.0 % patients in the LABD alone cohort and 22 % patients in LABA+ICS cohort experiencing at least one non-COPD hospitalisation. High proportions of patients in both cohorts (LABD alone [78 %] and LABA+ICS [82 %]) had a prescription for a concurrent SABD.

### Adherence to LABD therapy and LABA+ICS up to 24 months

Less than half the patients (LAMA: 42 %; LABA: 34 % and LABA+ICS: 34 %) were adherent (MPR ≥ 80 %) to their index prescription up to 24 months following treatment initiation. The mean total treatment time used to annualise the costs was shorter for adherent patients (LAMA: 371.5 vs. 479.4 days, LABA: 298.9 vs. 428.8 days and LABA+ICS: 381.4 vs. 495.9 days) compared with non-adherent patients (p < 0.001 for comparison in each class), suggesting that adherent patients in this cohort had, on average, a quicker time to treatment discontinuation or treatment change (switch or addition of another maintenance therapy). However, during that total treatment time, adherent patients were more regularly taking their medications, as evidenced by a longer mean drug exposed time (LAMA: 354.3 vs. 218.0 days; LABA: 280.0 vs. 186.3 days; LABA+ICS: 359.9 vs, 215.3 days; p < 0.01 for each comparison) and a higher mean number of prescriptions (LAMA: 12.6 vs. 7.4 prescriptions; LABA: 9.9 vs. 6.3 prescriptions; LABA+ICS: 12.7 vs. 7.2 prescriptions) compared to non-adherent patients.

**Fig. 2:**
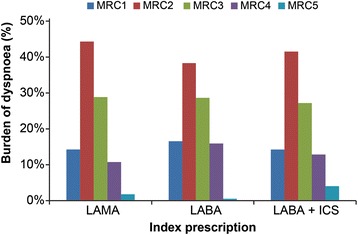
Burden of dyspnea among stable COPD patients by their index prescription. MRC-Medical Research Council dyspnea score, LAMA-long-acting muscarinic antagonist, LABA-long-acting beta2-agonist

Patient demographics and disease burden over 24 months follow-up by adherence to initial LABD therapy are presented in Table 3. Adherent patients tended to be slightly older and have symptoms of dyspnea, as defined by MRC scores (MRC ≥ 3) compared with non-adherent patients. Among adherent patients with a known value for MRC, 41 % starting on LAMA, 45 % starting on LABA and 44 % starting on LABA+ICS had clinically significant dyspnea (MRC ≥ 3) based on a recording closest to 24 months or last prescription (Fig. 2). The proportion of non-adherent patients who had a similar recording of clinically significant dyspnea was comparable. Adherent patients also trended towards having a higher rate of severe exacerbations during follow-up and appeared to have a shorter time to first moderate or severe exacerbation compared with non-adherent patients.

**Table 3:** Patient demographics and disease burden over 24 months follow-up by adherence to initial LABD therapy

	LAMA		LABA		LABA+ICS	
	MPR > = 80 %	MPR < 80 %	MPR > = 80 %	MPR < 80 %	MPR > = 80 %	MPR < 80 %
	(*N* = 2460)	(*N* = 3341)	(*N* = 355)	(*N* = 678)	(*N* = 2630)	(*N* = 5102)
Gender, male (%)	58	56	50	53	52	53
Age at index date, Mean (SD)	70.1 (10.0)	68.5 (10.5)	70.0 (10.2)	67.4 (10.6)	69.6 (10.7)	67.6 (11.6)
Comorbidities, Mean CCI (SD)^a^	0.3 (0.7)	0.3 (0.7)	0.3 (0.7)	0.2 (0.6)	0.2 (0.6)	0.2 (0.7)
Baseline BMI kg/m^2^, Mean (SD)	27.0 (6.2)	27.5 (6.2)	27.4 (6.0)	27.9 (6.0)	27.2 (6.4)	27.8 (6.4)
Dyspnea (%)^b^						
MRC < 3	59	60	55	62	56	60
MRC ≥ 3	41	40	45	38	44	40
Any exacerbations (Moderate to severe)^c^						
Rate per PY (95 % CI)	0.44 (0.41-0.47)	0.40 (0.38-0.42)	0.48 (0.41-0.57)	0.43 (0.39-0.48)	0.71 (0.68-0.74)	0.57 (0.55-0.59)
Mean Time to first event, months (SD)	6.7 (5.9)	7.8 (5.9)	6.7 (6.5)	7.7 (5.9)	6.5 (5.8)	7.6 (5.9)
Hospitalized (Severe) COPD exacerbations						
Rate per PY (95 % CI)	0.22 (0.21-0.24)	0.13 (0.12-0.14)	0.19 (0.14-0.24)	0.10 (0.08-0.12)	0.23 (0.22-0.25)	0.15 (0.14-0.16)
Mean Time to first event, months (SD)	8.2 (6.6)	9.9 (6.8)	6.7 (6.4)	9.2 (6.2)	8.5 (6.8)	9.4 (6.6)
Non-COPD hospitalizations						
Rate per PY (95 % CI)	0.34 (0.32-0.36)	0.39 (0.37-0.41)	0.31 (0.25-0.38)	0.37 (0.33-0.42)	0.38 (0.36-0.41)	0.47 (0.45-0.48)

CI, confidence interval; CCI, Charlson Comorbidity Index; COPD, chronic obstructive pulmonary disease; ICS, inhaled corticosteroid; LABA, long-acting beta2-agonist; LABD, long-acting bronchodilator; LAMA, long-acting muscarinic antagonist; MRC, Medical Research Council dyspnea scale 1-5; FEV1, Forced expiratory volume; PY, person years; SD, standard deviation

^a^Charlson Comorbidity Index score calculated based on co-morbidities recorded any time in the patient history up until the index date

^b^Percentages represent distribution in patients with a known value. Dyspnea data unknown for *N* = 1159 (47 %) LAMA users with MPR > = 80 % and *N* = 1093 (33 %) with MPR <80 %; *N* = 198 (56 %) of LABA users with MPR > = 80 % and *N* = 281 (41 %) with MPR <80 %; *N* = 1160 (44 %) LABA+ICS users with MPR > = 80 % and *N* = 1646 (32 %) with MPR <80 %

^c^Health care resource use and exacerbation data during the time period of index medication to the final prescription used in the MPR calculation (over 24 months of follow- up)

### Predictors of resource use

A total of six proportional hazard models were analysed, one for each treatment class (LAMA, LABA and LABA+ICS) for the two endpoints, time to first moderate-severe COPD exacerbation and time to first severe exacerbation. Each model resulted in slightly differing sets of predictor variables, but overall, prior history of exacerbations (0 vs 1 and 0 vs ≥ 2) and prior history of non-COPD hospitalizations (0 vs 1 and 0 vs ≥ 2) were the most consistent predictors in three of the six models each. Adherence and SABD use (<4 vs ≥ 4 prescriptions in the previous year) also were significant predictors of time to first severe exacerbation among patients that initiated an LABA+ICS (Table 4).

**Table 4:** Predictors of time to first moderate to severe exacerbation and time to first severe exacerbation by initial LABD therapy

	Hazard ratio (95 % CI)		
	LAMA	LABA	LABA+ICS
Time to first moderate-severe exacerbation			
Age (years)	NS	NS	1.01 (1.00-1.02)
Gender (Male vs Female)	0.59 (0.42-0.83)	NS	NS
COPD incident case (Yes vs No)	2.08 (1.05-4.13)	NS	NS
Number of moderate to severe exacerbations at baseline			
1 vs 0	2.11 (1.43-3.11)	NS	1.78 (1.41-2.25)
≥2 vs 0	5.00 (3.21-7.79)	NS	3.41 (2.73-4.26)
Number of non-COPD hospitalisations at baseline			
1 vs 0	NS	1.19 (0.40-3.52)	NS
≥2 vs 0	NS	15.87 (4.80-52.52)	NS
Time to first severe exacerbation			
MPR (≥80 % vs <80 %)	NS	NS	1.60 (1.17-2.19)
Age (years)	NS	NS	1.02 (1.01-1.04)
GP visits	NS	NS	1.01 (1.00-1.01)
SABD ≥4 vs 0	NS	NS	1.39 (1.02-1.89)
Number of moderate to severe exacerbations at baseline			
1 vs 0	1.65 (0.92-2.96)	NS	NS
≥2 vs 0	2.52 (1.29-4.91)	NS	NS
Number of non-COPD related hospitalisations at baseline			
1 vs 0	3.32 (1.75-6.30)	NS	1.81 (1.23-2.66)
≥2 vs 0	3.12 (1.51-6.45)	NS	1.71 (1.08-2.70)

NS, Not significant; CI, confidence interval; COPD, chronic obstructive pulmonary disease; ICS, inhaled corticosteroid; LABA, long-acting beta2-agonist; LABD, long-acting bronchodilator; LAMA, long-acting muscarinic antagonist; GP, general practitioner, SABD, short acting bronchodilator

### Costs incurred up to 24 months based on adherence

**Fig. 3:**
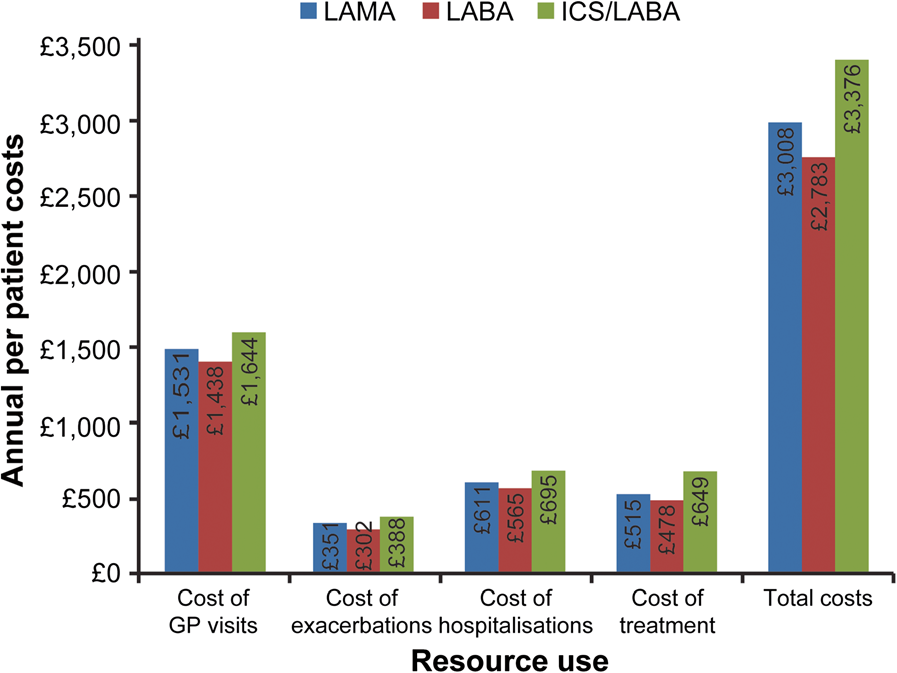
Annual per patient costs of resource use. Annual per patient costs over 24 months of resource use related to COPD among patients adherent to initial LABD therapy (MPR ≥ 80 %). COPD-chronic obstructive pulmonary disease; GP, general practitioner; ICS-inhaled corticosteroid; LABA-long-acting beta2-agonist; LABD-long-acting bronchodilator; LAMA-long-acting muscarinic antagonist; MPR, medication possession ratio

The total annual per patient cost among adherent patients was £3008 for LAMA initiators, £2783 for LABA initiators and £3376 for LABA+ICS initiators (Fig. 3). The corresponding estimates for the non-adherent cohort were significantly lower at £2526, £2373 and £2816, respectively (*p* < 0.0001 for all three comparisons). Approximately half of these costs comprised GP interactions and a fifth was contributed by non-COPD hospitalisation. Slightly over 10 % of costs were attributable to exacerbations, with the remainder accounted for by medications. This distribution of the cost components was similar, irrespective of index medication or adherence status.

## Discussion

This study estimated the symptomatic and cost burden among patients with COPD initiated on LABD alone or LABA+ICS treatment and explored the impact of adherence on healthcare resource use and costs. Results indicated that the annual costs of COPD management were high but consistent with recent studies completed in this setting [10, 11]. GP visits accounted for nearly half of the total costs, substantially more than severe exacerbations, medications or non-COPD hospitalisations. This trend was similar for all index medication classes regardless of adherence. Among those with a recorded value for MRC during follow-up, 40–50 % had high breathlessness at treatment initiation which was also observed during follow-up, even among patients adherent to their medications. This may possibly indicate insufficiency in the current treatment options to manage symptoms, a need for better assessment of symptoms including managing comorbidities which can contribute to dyspnea, or improved communication between the patient and healthcare professional about the impact of their COPD. Addressing this patient need is essential as others studies have shown that dyspnea is an important factor leading to disability among COPD patients [25].

The exacerbation rate in the year prior to initiation of maintenance therapy may guide treatment choices, as patients initiating on LABA+ICS therapy had a higher rate of moderate-severe exacerbations in the previous year compared with those initiating on a bronchodilator alone. This is in line with guidelines and literature which suggest that ICS in combination with bronchodilators is appropriate for patients with exacerbations and bronchodilators for symptomatic patients [1, 2]. However, our results also indicate that a high proportion of LABA+ICS initiators (55 %) do not have a history of exacerbations in the previous 12 months. Whilst these patients appear not to be treated in accordance with COPD guidelines, a high proportion of ICS users in our study had concomitant asthma (38 % vs 14 % for LABD) which may indicate ICS use consistent with the guidelines. Further, our finding of 55 % of ICS-users without prior exacerbation history is consistent with a recent study by Price and colleagues wherein 49 % of ICS users (alone or in combination) did not have an exacerbation history [26].

Among patients receiving LABD alone, no major differences were noted in patient demographics or prior resource use at baseline, suggesting that physicians did not favour one class of bronchodilators over another based on patient history. A propensity towards a particular choice of treatment may be driven by airflow limitation and the extent of symptoms, however among those with this information recorded, no marked trend was observed.

This study found that less than half of COPD patients were adherent to their index medication, consistent with a previous study conducted in CPRD [16]. Whilst the study by Wurst and colleagues [16] included newly diagnosed COPD patients, our study focussed on COPD patients initiating their first maintenance treatment, highlighting that non-adherence to medication is a challenge for COPD management regardless of the stage of disease. Future interventions focussed on improving adherence are needed in COPD to optimally manage patients. We also showed that the annual COPD management costs in adherent patients were higher that non-adherent patients for all three treatment classes. This is counterintuitive and inconsistent with other studies in the COPD literature, which found lower costs among adherent patients [27–29]. We hypothesize that adherent patients in our study had more severe disease, as evidenced by a shorter mean total treatment time (i.e. quicker time to treatment modification) and a trend toward more frequent hospitalisations for COPD and non-COPD reasons. This hypothesis was partially confirmed by multivariate analysis wherein history of exacerbations was the most consistent predictor of incidence of future exacerbations and thereby costs. Other variables associated with severity such as lung function and MRC were not significant in the predictive models, but with high proportions of missing data it is difficult to predict their true impact.

This study has several limitations. A large proportion of patients were missing data for the MRC dyspnea scale and GOLD stages of airflow obstruction suggesting that they were either not clinically evaluated for these by the GP or that they were evaluated by another health care provider and the data were not captured in the CPRD record. We identified only a small proportion of patients treated with a combination of LAMA and LABA in separate inhalers, and thus this class was not explored further in terms of adherence and disease burden. With recent launches of combination bronchodilators, the treatment pattern may change over time and may limit the long term applicability of this study to UK clinical practice. Further, the generalizability of these findings outside the UK may be limited. Lastly, CPRD captures information on prescribed rather than dispensed prescriptions and there was no further verification that patients were actually taking their medications as prescribed. Previous audits of electronic medical records have demonstrated a relatively high concordance of dispensing to prescribing in the UK (99.7 % of prescriptions tracked were recorded by electronic patient records during a month) [30].

## Conclusion

Among patients initiating maintenance treatment for COPD, adherence to the index medication was low. Many adherent patients were symptomatic across all LABD classes studied. In our setting, the COPD management costs were high among adherent and non-adherent patients with GP interactions contributing the most. Real world studies are required to inform interventions to address unmet needs, including impact of patient engagement tools on adherence, integrated management of comorbid conditions, and optimizing treatment pathways to improve COPD symptom burden and outcomes.

## Abbreviations

LABD: Long-acting bronchodilator
LAMA: Long-acting muscarinic antagonist
LABA: Long-acting beta2-agonist
COPD: Chronic obstructive pulmonary disease
ICS: Inhaled corticosteroid
SABD: Short-acting bronchodilators
COPD: Global Initiative for Chronic Obstructive Lung Disease
CPRD: Clinical Practice Research Datalink
BMI: Body mass index
MRC: Medical Research Council
MPR: Medication Possession Ratio
NHS: National Health Service
HRG: Healthcare Resource Group
A&E: Accident and Emergency
BNF 65: British National Formulary 65
GOLD: Global Initiative for Chronic Obstructive Lung Disease
GP: General Practioner
PCA: Prescription cost analysis
PSSRU: Personal Social Services Research Unit
NICE: National Institute for Health and Care Excellence
